# Extensively Drug-Resistant *Shigella flexneri* 2a, California, USA, 2022

**DOI:** 10.3201/eid2907.230465

**Published:** 2023-07

**Authors:** J.R. Caldera, Shangxin Yang, Daniel Z. Uslan

**Affiliations:** David Geffen School of Medicine at University of California, Los Angeles, California, USA

**Keywords:** Shigella, extensively drug resistant, XDR, whole-genome sequencing, WGS, next-generation sequencing, NGS, bacteria, California, United States

## Abstract

In Los Angeles, California, USA, persistent, refractory shigellosis was diagnosed in an immunocompetent man who has sex with men. Whole-genome sequencing augmented phenotypic antimicrobial susceptibility testing to comprehensively profile bacterial drug resistance and appropriately guide therapy and clear the infection.

Shigellosis is an infectious diarrheal illness traditionally associated with contaminated food and water (*1*). Recent reports, however, have indicated increased transmission through sexual activity, including direct (anal/oral sex) and indirect (use of contaminated objects such as sex toys) sexual contact (*2*). Several outbreaks of extensively drug-resistant (XDR) *Shigella* spp. among men who have sex with men (MSM) have been described (*3*–*5*).

Recently, the emergence of sexually transmitted XDR *Shigella flexneri* serotype 2a was reported among gay, bisexual, and other MSM in England (*4*). The study by the UK Health Security Agency characterized *S. flexneri* isolates harboring *bla*_CTX-M-27_ and identified the 2 phylogenetically related clusters, each composed of isolates within 10 single-nucleotide polymorphisms (SNPs). Long-read sequencing elucidated the genomic location of the resistance determinant, and plasmid similarities with XDR *Shigella sonnei* from a current outbreak supported interspecies horizontal acquisition of drug resistance. Those findings underscore the substantial threat of mobilizable genetic elements and their role in propagating antimicrobial resistance (AMR). We describe a case of extensively drug-resistant *S. flexneri* infection in a 36-year-old man in California, USA. 

The patient was an HIV-negative man who has sex with men and who had a history of shigellosis (*S. flexneri* type 1) 1.5 years earlier and mpox 4 months earlier. He sought hospital care for fever, abdominal pain, nonbloody watery diarrhea, and mild leukopenia. A fecal culture grew *S. flexneri* type 2, which was treated with ciprofloxacin for 4 days. Antimicrobial susceptibility testing (AST) was not requested. The patient’s sign/symptoms and leukocyte count slowly improved, although he continued to have loose bowel movements with mucous. He was referred for outpatient infectious diseases consultation. At that time, the patient’s history included frequent anonymous sexual encounters, typically including performing and receiving fellatio, analingus, and insertive anal intercourse; he stated he always wore condoms for intercourse but did not use barrier protection for fellatio or analingus. Testing for other sexually transmitted infections (including gonorrhea, chlamydia, HIV infection, and syphilis) produced negative results. Because his signs/symptoms were persistent, we sent repeat fecal samples to the UCLA Health Clinical Microbiology Laboratory for bacteria and parasites studies, including the BD Max Enteric Bacterial Panel PCR test (BD, https://www.bd.com), and results were again positive for *Shigella* spp. The patient elected to undergo observation only, and no additional treatment was provided.

Subsequent fecal samples sent several weeks later were again positive for *S. flexneri*, and the Los Angeles County Public Health Laboratory serotyped the cultured isolate as 2a. AST of the most recent isolate identified extensive AMR (Table). The patient was asymptomatic at that time. Because of a lack of other oral antimicrobial options, on the basis of AST results we chose minocycline for initial treatment to avoid parenteral treatment and the associated risks, solely to reduce bacterial shedding.

**Table:** AST profile for extensively drug-resistant *Shigella flexneri* 2a isolate UCLA_1207, 2023, from patient in California, USA*

Drug	MIC, mcg/mL	Interpretation	Detected AMR gene
Amoxicillin + clavulanate	32	Resistant†	*bla*_OXA-1_, *bla*_CTX-M-15_
Ampicillin	>32	Resistant†	*bla*_OXA-1_, *bla*_CTX-M-15_
Azithromycin	32	Resistant	*mph(A)*
Cefazolin	>32	Resistant†	*bla*_OXA-1_, *bla*_CTX-M-15_
Cefepime	4	Susceptible, dose dependent†	*bla*_CTX-M-15_
Ceftazidime	4	Susceptible†	*bla*_CTX-M-15_
Ceftazidime/avibactam	<2	Susceptible†	None
Ceftriaxone	64	Resistant†	*bla*_CTX-M-15_
Ciprofloxacin	>4	Resistant	*qnrS1, gyrA* D87N+S83L
Doxycycline	>16	Resistant†	*tet(B)*
Ertapenem	<0.25	Susceptible†	None
Imipenem	<1	Susceptible†	None
Levofloxacin	>8	Resistant†	*qnrS1, gyrA* D87N+S83L
Meropenem	<0.25	Susceptible†	None
Minocycline	4	Susceptible†	*tet(B)*
Piperacillin + tazobactam	<8	Susceptible†	*bla*_OXA-1_, *bla*_CTX-M-15_
Trimethoprim/sulfamethoxazole	>4/80	Resistant	*dfrA17, sul1*
Fosfomycin	Not performed‡	Not performed‡	Not applicable

*AMR, antimicrobial resistance; AST, antimicrobial susceptibility testing. †AST not initially included in primary report and performed and reported at physician request. ‡AST not performed because of lack of interpretive guidelines by the Clinical and Laboratory Standards Institute.

Ultimately, inconsistencies in AST results together with concerns over the XDR phenotype prompted whole-genome sequencing (WGS) of the isolate with the Illumina MiSeq platform (Illumina, https://www.illumina.com). K-mer–based phylogenic analysis (KmerFinder version 3.2, https://cge.food.dtu.dk/services/KmerFinder) identified *S. flexneri* 2a strain 981 as the most closely related reference, with 99.70% genome coverage and 98.20% pairwise identity (*6*–*8*). We performed further SNP analysis for microbial relatedness against a previously sequenced multidrug-resistant *S. flexneri* 2a isolate (UCLA 2020, which did not carry a CTX-M gene) from man who has sex with men residing in Riverside, California, along with the publicly available sequences from the United Kingdom study, which were all serotype 2a with plasmid-borne *bla*_CTX-M-27_ (*4**,**9*). Analysis revealed that the 2 California isolates are closely related to the UK *S. flexneri* strains, which contain <200 SNPs (Figure).

**Figure:**
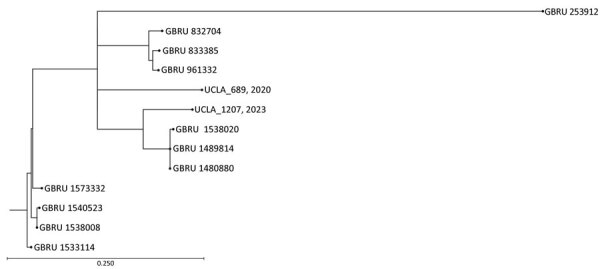
Maximum-likelihood single-nucleotide polymorphism tree of *Shigella flexneri* strain 2a isolates from California, USA (UCLA_689, 2020, and UCLA_1207, 2023), and the United Kingdom. Analysis was performed by using CLC Genomics Workbench version 22.0.2 (https://www.qiagen.com) single-nucleotide polymorphism analysis for microbial relatedness workflow and using reference strain *Shigella flexneri* 2a strain 981. GBRU, UK Gastrointestinal Bacterial Reference Unit; UCLA, University of California Los Angeles. Scale bar indicates nucleotide substitutions per 100 sites.

AMR gene analysis performed by using ResFinder version 4.0 (https://cge.food.dtu.dk/services/ResFinder) identified a compendium of AMR genes that not only corroborated the phenotypic AST results but also reconciled the ambiguous or discrepant categorical results within classes of antimicrobials (Table). Specifically, detection of *bla*_CTX-M-15_ indicated that an extended-spectrum β-lactamase enzyme had been acquired and suggested resistance to all cephalosporins, which contrasts with the elevated, yet susceptible, MIC for ceftazidime and cefepime. Detection of *tet(B)* indicated that resistance to tetracyclines is not limited to doxycycline but also includes minocycline, again in contrast with the elevated, yet susceptible, MIC determined by AST. Moreover, the absence of detectable fosfomycin resistance–conferring gene(s) provided confidence that fosfomycin was appropriate for patient management.

Thus, with information from the WGS-based genomic characterization, we replaced minocycline treatment with fosfomycin (3 g by mouth every other day for 3 doses). Three weeks after completing treatment with fosfomycin, the patient’s PCR result was negative, indicating clearance of *Shigella*.

In summary, persistent shigellosis caused by XDR *S. flexneri* was successfully treated with fosfomycin, as guided by WGS. Moreover, we demonstrate the genetic relatedness of 2 isolates from California to an ongoing outbreak in Europe, although with different CTX-M genes. This case illustrates the clinical value of WGS not only for providing a more comprehensive bacterial drug resistance profile but also for tracking the global epidemiology of clinically important organisms.
